# Cue-Induced Ethanol Seeking in *Drosophila melanogaster* Is Dose-Dependent

**DOI:** 10.3389/fphys.2018.00438

**Published:** 2018-04-23

**Authors:** Kavin M. Nunez, Reza Azanchi, Karla R. Kaun

**Affiliations:** ^1^Molecular Pharmacology and Physiology Graduate Program, Brown University, Providence, RI, United States; ^2^Department of Neuroscience, Brown University, Providence, RI, United States

**Keywords:** *Drosophila*, ethanol, alcohol-use disorder, memory, addiction, reward

## Abstract

Alcohol use disorder generates devastating social, medical and economic burdens, making it a major global health issue. The persistent nature of memories associated with intoxication experiences often induces cravings and triggers relapse in recovering individuals. Despite recent advances, the neural and molecular mechanisms underlying these memories are complex and not well understood. This makes finding effective pharmacological targets challenging. The investigation of persistent alcohol-associated memories in the fruit fly, *Drosophila melanogaster*, presents a unique opportunity to gain a comprehensive understanding of the memories for ethanol reward at the level of genes, molecules, neurons and circuits. Here we characterize the dose-dependent nature of ethanol on the expression of memory for an intoxication experience. We report that the concentration of ethanol, number of ethanol exposures, length of ethanol exposures, and timing between ethanol exposures are critical in determining whether ethanol is perceived as aversive or appetitive, and in how long the memory for the intoxicating properties of ethanol last. Our study highlights that fruit flies display both acute and persistent memories for ethanol-conditioned odor cues, and that a combination of parameters that determine the intoxication state of the fly influence the seemingly complex retention and expression of memories associated with intoxication. Our thorough behavioral characterization provides the opportunity to interrogate the biological underpinnings of these observed preference differences in future studies.

## Introduction

A critical component of the recurring nature of alcohol use disorder (AUD) involves the cravings elicited by ethanol exposures (priming doses), cues, and stress (Ludwig and Wikler, 1974; Hodgson et al., 1979; Le et al., 1998, 1999; Gass and Olive, 2007). Cue reactivity to ethanol-conditioned cues is an indicator of urges, predictor of relapse, and used to monitor putative treatments (Niaura et al., 1988; Monti et al., 1993; Rohsenow et al., 1994; Sayette et al., 1994; McGeary et al., 2006; Witteman et al., 2015). Although these studies recognize the importance of cue reactivity, the biological underpinnings of cue reactivity are not fully delineated. Moreover, in a natural environment cravings may be elicited in a more complex manner. Comprehensively understanding how ethanol-associated cue memories are formed and expressed may provide valuable insight to understanding the recurring nature of AUD.

Model systems provide the opportunity to characterize the biology underlying cue-induced cravings. Memory for ethanol associated cues are demonstrated in a wide range of species, from nematodes to primates (Smith et al., 1984; Reid et al., 1985; Bozarth, 1990; Colombo et al., 1990; Suzuki et al., 1992; Lee et al., 2009; Mathur et al., 2011). Although rodent models are the predominant model organism used to study cue-induced ethanol seeking, *Drosophila melanogaster* offer distinct advantages in defining the biology of cue-induced ethanol seeking. Not only do the genetic tools available in *Drosophila* permit precise spatial and temporal control of gene expression (Venken and Bellen, 2005; del Valle Rodriguez et al., 2011), but *Drosophila* show persistent preference for an odor cue previously associated with ethanol intoxication (Kaun et al., 2011). This provides the ability to define precise circuit motifs, and the accompanying molecular mechanisms required for behavior. However, before leveraging these tools in *Drosophila*, extensive characterization of factors impacting cue-induced ethanol preference is required. This is a critical step in avoiding mis- or overinterpretation of the results derived from future mechanistic studies.

In humans, dose-response relationships for addictive substances such as ethanol follow an inverted ‘U’ shaped curve where the ascending slope builds towards a peak appetitive response associated with reward and euphoria, and the descending slope depicts aversive states of dysphoria, anxiety and withdrawal (Van Etten et al., 1995; Tomie et al., 1998; Uhl et al., 2014). Similarly, in rodent models the dose and duration of ethanol intoxication affects the valence and strength of memories for a cue-associated experience (Bozarth, 1990; Risinger and Oakes, 1996; Shimizu et al., 2015). Although sensitivity and tolerance to ethanol have been well characterized in *Drosophila*, less is understood about the behavioral intricacies underlying the appetitive and aversive properties of the intoxication experience. We hypothesized that the extent and timing of intoxication would impact an animal’s preference for cues associated with alcohol. Using a conditioned preference assay to test preference for an olfactory cue previously associated with ethanol intoxication, we characterized how intoxication affects valence and magnitude of cue memory for intoxication in *Drosophila.* We also characterized the administered dose concentration, duration, number of exposure sessions, latency between exposures, and time until testing to understand how these variables shape preference. This extensive characterization provides a framework within which future investigations will inform behavioral and pharmacological interventions to inhibit cue-induced cravings and relapse.

## Materials and Methods

### Stocks and Conditions

Canton-S (CS) wild-type flies were used for all experiments. Flies were reared at 25°C and 70% humidity on a 12:12 Light:Dark (L:D) cycle with lights on at 8:00am. Flies were raised in 9.5 cm (height) × 2.5 cm (diameter) polypropylene vials on standard Bloomington cornmeal, molasses, and yeast media. Groups of 50 male flies were collected 0–1 days after eclosion under CO_2_-induced anesthesia. Flies were given 2 days to recover from the CO_2_ anesthesia, stored in groups of 50 in food vials at 25°C and 70%, on the same 12:12 L:D cycle. Behavior experiments were initiated when flies were 3–5 days old (adult flies). Importantly, because flies were sacrificed following each test, different groups of flies were used for each experiment reported here.

### Environmental Conditions for Behavior Experiments

**FIGURE 1:**
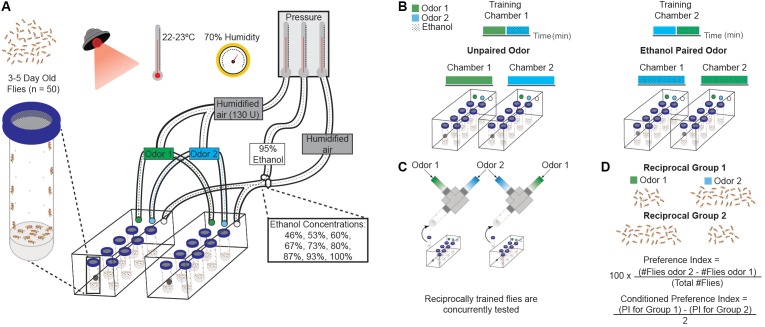
Ethanol conditioning assay. **(A)** Schematic of behavioral training apparatus and fly training conditions. Behavioral training is done under red light, with temperature and humidity controlled throughout. Male flies (*n* = 50) are transferred into perforated vials with 1 mL of 1% agar and acclimated in the behavioral boxes for 15 min with 130 U of humidified air. Air pressure for ethanol and humidified air is controlled to adjust the administered dose. A passive vacuum is present within the behavioral boxes for air/odorant removal. **(B)** Olfactory-cues are paired with vaporized ethanol during training. Varying ethanol concentrations are achieved by adjusting the flow rates of 95% vaporized ethanol and humidified air. Flies are trained with two neutral or appetitive odors: one unpaired with the ethanol and the other paired with ethanol. A reciprocal paradigm is used to control for innate odor preference. **(C)** Flies are tested with the two odor cues in a Y-maze (displayed above). Reciprocally trained flies are tested concurrently. **(D)** A preference index (PI) is calculated by subtracting the flies that move towards odor one from the flies that move towards odor two, dividing the resultant number by the total number of flies, and multiplying that number by 100. A conditioned preference index (CPI) is calculated by subtracting the preference index for odor two from the preference index for odor one and dividing the resulting number by two. A positive CPI value indicates preference, while a negative value indicates aversion. Each individual sample (N) is composed of 100 flies.

All behavioral experiments were based on the original cue-induced ethanol seeking or ‘ethanol reward memory’ behavior paradigm outlined in Kaun et al. (2011), and described in more detail below (also see **Figure 1**): For all behavioral experiments, flies were not food- or water-deprived prior to training. Throughout training and testing periods, flies were kept in a dark-room under red-light at 22–23°C and 70% humidity. The temperature was controlled with an oil-filled radiator (DeLonghi TRD0715T, Dubuque, IA, United States) and humidity controlled with a warm-mist humidifier (Vicks V745A, Procter & Gamble, San Ramon, CA, United States). Temperature and humidity were constantly monitored throughout training and testing to ensure consistent conditions across experiments.

### Ethanol-Odor Training

Flies were trained in perforated vials (2.5 cm diameter and 9.5 cm height) containing 1 mL of 1% agar. Vials contained 64 evenly-spaced perforations (∼1 cm spacing throughout 74 cm^2^ surface area of vial without top and bottom circular surfaces) and a mesh lid to facilitate uniform distribution of ethanol within the vials. Vials were placed into a test-tube holder in a 30 cm length × 15 cm height × 15 cm width training chamber (Aladin Enterprises, Inc., San Francisco, CA, United States). The training chamber contained three nozzles to allow for air/odorants/ethanol to stream in and one exhaust nozzle for waste. Flies were given a 10–15 min acclimation period within the training chamber prior to the start of experiments.

Humidified air was bubbled through 95% ethanol to vaporize ethanol, which was then combined with humidified air in various proportions. Humidified air was streamed over odors placed in a 2.5 cm diameter and 13 cm height cylinder at a flow rate of 130 U for training and 100 U for tests (where 100 U is equal to 1.7 L/min at room temperature). Odor flow rates were decreased during the test to ensure that the odors do not intermix in the Y-ends, allowing the flies to sufficiently discriminate between the two different odors during the choice test. The odors we used were either 3 mL iso-amyl alcohol (1:36 in mineral oil) or a mixture of 2 mL ethyl acetate (1:36 in mineral oil) and 1 mL acetic acid (1:400 in mineral oil). Odors were replaced daily to reduce any effects of odor evaporation. Humidified air (130 U) was flowed through training boxes during acclimation and rest periods.

Reciprocal odor training was performed to account for any inherent odor preference. Unless stated otherwise, a training period generally consisted of exposure to odor cue 1, followed by exposure to odor 2 with vaporized ethanol. A separate group of flies was simultaneously trained with exposure to odor 2, then odor 1 with vaporized ethanol (**Figure 1**). These training periods varied in exposure duration, number of training sessions and rest periods throughout this study. Vials of flies from Group 1 and Group 2 were paired according to placement in the training chamber and tested simultaneously. Vials tested simultaneously were averaged together to get a conditioned preference index.

### Conditioned Odor Preference Test

The testing chamber was a 6 cm cube with a mesh Y-maze in the middle (Aladin Enterprises, Inc., San Francisco, CA, United States). During testing periods odors were streamed in through opposite arms of the Y (each 6 cm). Vials of flies were placed at the base of the Y and flies climbed up the mesh cylinder, where they chose between opposing arms of the Y that were capped with collection vials (2.5 cm diameter, 9.5 cm height). After 2 min, vials were removed, plugged, and covered with tape to trap flies within the collection tubes. The number of flies that moved into the odor 1 and odor 2 vials were counted after vials were frozen at either -20°C for 1 h, or -80°C for 20 min. Preference index (PI for each group was calculated as [(# flies in odor 1 vial – # flies in odor 2 vial) / total # flies] ^∗^ 100. A conditioned performance index (CPI) for conditioned odor preference or aversion was calculated by subtracting the PI for reciprocal group 2 from the PI of reciprocal group 1 and dividing by 2.

Memory was tested either 30 min or 24 h post-training. For all flies tested 24 h post-training, yeast pellets were carefully added to the training vials 1 h post-training to ensure flies did not become food deprived prior to testing. For characterization experiments that took place across several days, flies were trained on food containing 10 g yeast, 10 g sugar and 4 g agar boiled in 200 mL water.

### Statistical Analysis

All conditioned preference indexes are plotted as bars representing means +/- standard error. Individual data points plotted represent *N* = 1 (∼100 flies) calculated by averaging preference indexes per reciprocally trained groups (∼50 flies), accounting for any innate odor preference. On all data plotted here, CPIs of zero depict no memory formation, CPIs greater than zero depict appetitive memory, and CPIs less than zero depict aversive memory (see ‘Test for Conditioned Odor Preference’ above and **Figure 1** for a more detailed explanation).

Statistical analysis was performed using JMP^®^ Pro 13.2.0 licensed to Brown University. Comparisons were made between Preference Indexes for each reciprocal group within a condition. This tested whether preference for the paired odor was significantly different than for the unpaired odor, while controlling for innate preferences for either odor. All data conformed to equal sample sizes and the assumption of normality (Shapiro-Wilk test). The data between different doses did not consistently meet the assumption of equal variances using a Brown-Forsythe test, thus, a test that permits comparisons between groups with unequal variances was deemed necessary. Data was considered statistically significant when *p* < 0.05 using a Welch’s unequal variances two-tailed *t*-test. No data were removed as outliers in order to provide an accurate depiction of variability within the data.

## Results

### Ethanol Dose Influences Valence of Cue Memories

In *Drosophila*, ethanol dose affects ethanol-induced increases in locomotion, sedation, tolerance, and consumption (Moore et al., 1998; Scholz et al., 2000; Singh and Heberlein, 2000; Wolf et al., 2002; Berger et al., 2004; Devineni and Heberlein, 2009; Kaun et al., 2012). We previously showed that three doses of 53% vaporized ethanol (approximately 6mM or 0.025 g/dl body alcohol content per dose) induces an aversive memory shortly after exposure, and an appetitive memory 18 h to 7 days after exposure (Kaun et al., 2011). We sought to understand how changing the parameters of odor-ethanol pairings affected expression of memory for the odor cue.

**FIGURE 2:**
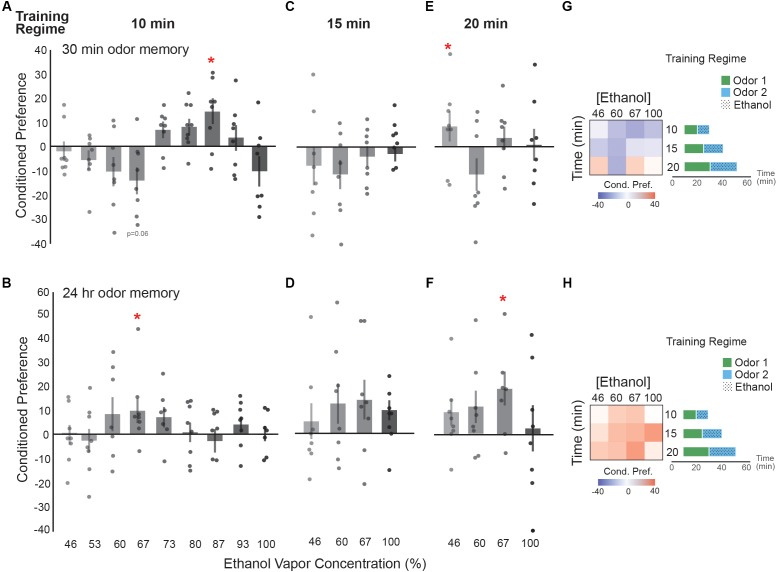
Ethanol preference is dose-dependent. **(A)** Distinct groups of flies were exposed to ethanol at increasing concentrations and sacrificed prior to calculating a preference score. Flies receiving a 67% dose of ethanol vapor showed a trending aversive memory [*t*(13.75) = 2.08, *p* = 0.06] whereas flies receiving a 87% ethanol vapor dose show a significant appetitive memory [*t*(13.81) = -2.34, *p* = 0.04]. **(B)** Flies receiving a 67% ethanol vapor dose show a significant appetitive memory [*t*(14.00) = -2.56, *p* = 0.02]. **(C)** A non-significant trend toward aversive preferences occurs at 30 min post-training when exposure duration is increased to 15 min. **(D)** A non-significant trend toward appetitive ethanol preference is displayed at 24 h with a 15 min exposure duration. **(E)** Increasing the exposure duration to 20 min results in significant appetitive memory at 30 min post-training with 46% ethanol [*t*(14.00) = 2.74, *p* = 0.02]. **(F)** Exposure duration of 20 min results in a long-term appetitive ethanol preference with 67% ethanol [*t*(11.96) = -3.51, *p* = 0.004]. **(G)** Heat map summary of 30 min ethanol preference across different exposure durations suggesting that single 10 and 15 min exposure trainings trend toward an aversive (blue) response to ethanol at 30 min post-training. Schematics of single exposure training paradigm are depicted. **(H)** Heat map summary suggests a trend towards appetitive (red) ethanol preference at 24 h. Bars represent mean +/- standard error. N∼8 per group where individual data points represent *N* = 1 (∼100 flies) CPI. ^∗^*p* < 0.05.

We first characterized single exposure trainings across ethanol concentrations and exposure durations (10, 15, and 20 min), followed by testing for preference at 30 min and 24 h post-training. Of note, most of the single-dose characterizations did not display statistical significance, so we focus on observed trends to guide the following experiments and interpretation in our study. When testing preference 30 min after a single 10 min exposure there is a significant appetitive memory when training with an ethanol concentration of 87% (**Figure 2A**), however, this memory did not last 24 h (**Figure 2B**). Interestingly, we observed that a dose that induced a trend toward aversive memory 30 min after training (67% ethanol, **Figure 2A**), resulted in a lasting appetitive memory 24 h later (**Figure 2B**). This lasting appetitive dose for ethanol corresponding to a single low-dose results in approximating 9 mM body ethanol concentration or 0.04 g/dl (corrected for baseline) (Kaun et al., 2011). Thus, we used 67% and the slightly lower 60% ethanol doses as a reference to try increasing the duration of ethanol exposure to 15 or 20 min. 46% and 100% were included as lower and upper limits accordingly.

Fifteen minute exposures showed a similar trend to 10 min exposures where seemingly aversive 30 min memories corresponded to 24 h appetitive memories, however, none of these results were statistically significant (**Figures 2C,D**). Training with 20 min exposures results in a significant appetitive preference at 46% ethanol 30 min but not 24 h after training, whereas a 67% concentration results in an appetitive preference 24 h but not 30 min after training (**Figures 2E,F**). Thus, although single ethanol exposures don’t produce a large conditioned preference score, the most notable observation from the data is that the subtle shift from aversive or neutral valence towards an appetitive preference 24 h later is consistent across many doses (**Figures 2G,H**).

### Binge-Like Intoxication Induced Short-Lived Appetitive Cue Memories

**FIGURE 3:**
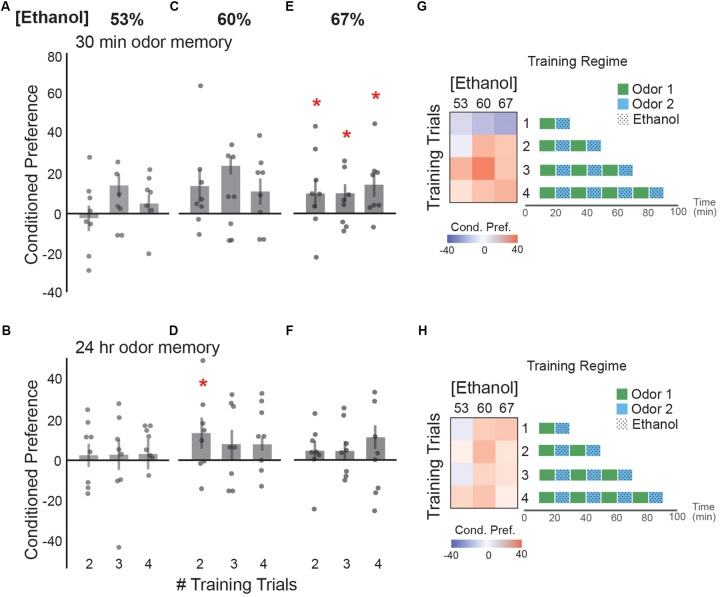
Binge-like alcohol-odor pairings results in an appetitive ethanol preference. No statistically significant ethanol preference is observed **(A)** 30 min post-training or **(B)** 24 h post-training with two, three, or four consecutive training trials of 53% ethanol. **(C)** Two [*t*(13.37) = -2.14, *p* = 0.051] or three [*t*(12.88) = -2.11, *p* = 0.054] consecutive doses of 60% ethanol trend towards an appetitive ethanol preference 30 min. **(D)** There is an observed 24 h ethanol preference following two, but not three or four consecutive doses of 60% ethanol [*t*(13.86) = -2.33, *p* = 0.04]. **(E)** Appetitive ethanol preference is observed at 30 min following two [*t*(13.89) = -2.26, *p* = 0.04], three [*t*(13.89) = -2.26, *p* = 0.04] or four [*t*(12.98) = -2.35, *p* = 0.03] consecutive doses of 67% ethanol. **(F)** No significant ethanol preferences are displayed 24 h following two, three, or four consecutive doses of 67% ethanol. **(G)** A heat map summary of the data from both the single exposure training and binge-like training demonstrates that binge-like training results in trend towards an appetitive ethanol preference at 30 min with multiple consecutive pairings. Schematic of the training paradigms are depicted. **(H)** A heat map summary of conditioned preference at 24 h post-training suggests very little lasting appetitive preference regardless of concentration and number of training trials. Bars represent mean +/- standard error. N∼8 per group where individual data points represent *N* = 1 (∼100 flies) CPI. ^∗^*p* < 0.05.

Repeated exposure to the same cue during intoxication strengthens the memory for that cue, making it a more salient predictor of ethanol reward (Tomie et al., 2002; Krank, 2003). Drinking norms observed in social environments often involve binge-consumption of ethanol, in which consecutive drinks are consumed before the effects of the first drink tapers. How this affects initial memory for cues associated with intoxication is, for the most part, unknown. We found 53% ethanol vapor did not significantly affect memory 30 min or 24 h after training (**Figures 3A,B**). Two, or three doses of 60% ethanol trended towards an appetitive memory 30 min after training (**Figure 3C**), which persisted 24 h after training following two but not three doses of 60% ethanol (**Figure 3D**). Interestingly, two, three or four consecutive doses of 67% ethanol induced a small but significant memory 30 min after training (**Figure 3E**). This memory did not persist 24 h after training (**Figure 3F**). Together, the trends in our data suggest that initially a single exposure of alcohol may result in an aversive memory whereas, two or more binge-like low-dose ethanol exposures trend towards a short-lived appetitive memory with few lasting effects (**Figures 3G,H**).

### Number of Spaced Ethanol Doses Determines Valence of Cue Memories

Long-lasting memory is induced by associations spaced by rest periods (Spreng et al., 2002; Commins et al., 2003). In the context of memories associated with alcohol intoxication, one might consume two or more glasses of wine over several hours. The wine may be consumed at a slow pace and consistently spaced over time, thus maintaining a mild euphoria throughout consumption. This consistent spacing doesn’t allow for inebriation to occur. Alternatively, it may be consumed more quickly and promote inebriation, rather than constant mild euphoria.

**FIGURE 4:**
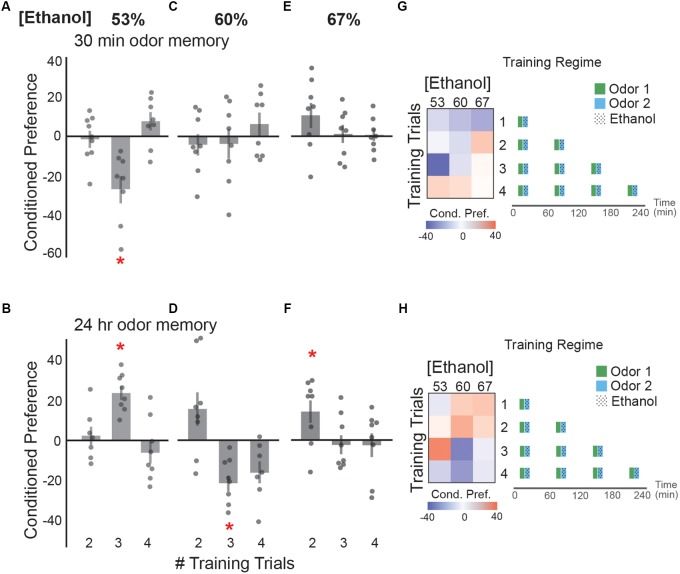
Preferences following spaced training depend on ethanol dose. **(A)** Three spaced training sessions of 53% ethanol concentration resulted in a significant aversive preference at 30 min [*t*(13.45) = 3.57, *p* = 0.003]. **(B)** The same training conditions induced a significant appetitive preference when tested at 24 h [*t*(12.58) = -6.57, *p* = 0.0001]. **(C)** Spaced training sessions with 60% ethanol concentration do not induce a preference at 30 min, but **(D)** result in a trend toward an appetitive memory after two trials [*t*(11.79) = -0.83, *p* = 0.09] and an aversive memory after three trials [*t*(11.86) = 3.92, *p* = 0.002] at 24 h post- training. **(E)** Spaced sessions with 67% ethanol concentration do not induce significant preference at 30 min, but **(F)** result in an appetitive ethanol preference after two trials [*t*(13.05) = -2.49, *p* = 0.03] at 24 h post-training. **(G)** Heat maps summarizing the 30 min preference relationships with training trial number and ethanol concentration suggest a complex dose relationship where an increase in the number of training trials and dose of ethanol trends towards a switch from appetitive (red) to aversive (blue) memory. Schematic of the training paradigms are depicted. **(H)** Inversely, heat maps displaying the preference trends at 24 h suggest that an increase in the number of training trials and dose of ethanol trends towards a switch from appetitive (red) to aversive (blue) memory. Bars represent mean +/- standard error. N∼8 per group where individual data points represent *N* = 1 (∼100 flies) CPI. ^∗^*p* < 0.05.

To test how spacing ethanol exposures over time affects cue memory, we exposed flies to two, three or four ethanol-odor pairings with a 50 min rest period in between pairings (**Figure 4**). This rest period was sufficient to decrease body ethanol concentration to ethanol-naive levels (Kaun et al., 2011). Three spaced pairings between an odor and 53% vaporized ethanol resulted in a significant aversive memory for an odor cue 30 min after training (**Figure 4A**), and appetitive memory 24 h after training (**Figure 4B**). Spaced-training with 60% ethanol vapor resulted in no 30 min memory (**Figure 4C**), but an appetitive memory trend after two pairings, and a significant aversive memory after three pairings (**Figure 4D**). Spaced training with 67% ethanol induced no 30 min memory (**Figure 4E**), but resulted in a significant appetitive memory after two training sessions (**Figure 4F**). Together, this data suggests that the strongest lasting appetitive response occurs after low dose exposures that include two spaced pairings with each dose approximating 8–9 mM (0.03–0.04 g/dl), or three spaced pairings of 6 mM (0.025 g/dl). Further, the trends suggest that too many ethanol exposures result in either the absence of 24 h memory or an aversive 24 h memory. This suggests that perhaps mild intoxication with sufficient rest to account for metabolism, rather than inebriation, is initially most appetitive. Additionally, reminiscent of memory after a single ethanol-odor pairing (**Figure 2**), conditions that trended toward short-term aversion also trended toward appetitive memory 24 h later (**Figures 4F,G**).

### Daily Ethanol Induces Long-Lasting Appetitive Cue Memories

**FIGURE 5:**
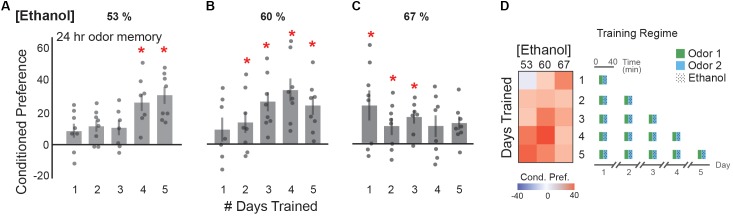
Single daily exposures result in an appetitive ethanol preference. **(A)** Appetitive ethanol preference at 24 h increases with the number of single daily training exposures with a 53% ethanol concentration [4 trials *t*(11.66) = -5.76, *p* = 0.0001; 5 trials *t*(13.01) = -4.70, *p* = 0.0004]. **(B)** Similarly, the number of daily exposures correlates with the magnitude of appetitive ethanol preference observed at 24 h with a 60% ethanol concentration [2 trials *t*(13.83) = -2.27, *p* = 0.04; 3 trials *t*(13.09) = -4.36, *p* = 0.0007, 4 trials *t*(8.42) = -5.36, *p* = 0.0006; 5 trials *t*(11.01) = -4.07, *p* = 0.002]. **(C)** Daily sessions of 67% ethanol concentration suggest saturated appetitive ethanol preference with greater than three training days, [1 trial *t*(9.82) = -2.95, *p* = 0.01; 2 trials *t*(10.33) = -2.43, *p* = 0.03, 3 trials *t*(11.43) = -4.27, *p* = 0.001]. **(D)** Heat map summaries suggest that the number of daily exposure trainings correlate with the observed appetitive preference at 24 h, with the strongest responses in the middle, characteristic of a U-shaped response. Schematic of the training regimes are depicted. Bars represent mean +/- standard error. N∼8 per group where individual data points represent *N* = 1 (∼100 flies) CPI. ^∗^*p* < 0.05.

Although a single early experience with ethanol can induce a lasting appetitive response (Warner and White, 2003), repeated daily ethanol consumption is more characteristic of consumption in modern society (Grant et al., 2017). Thus, we tested whether spacing single odor-intoxication pairings by 1 day induced a dose-dependent, lasting appetitive memory. We found that conditioned preference for an odor cue associated with 53 or 60% ethanol vapor generally increased as the number of training days increased (**Figures 5A,B**). A significant appetitive memory was observed at 4 and 5 days of training with 53% ethanol (**Figure 5A**), and after 2 days with 60% ethanol (**Figure 5B**). Increasing the number of days of training to 4 days with 60% ethanol appeared to increase the appetitive memory (**Figure 5B**). Training with 67% ethanol vapor resulted in a significant appetitive memory after 1 day of training, with a trend towards a decrease in preference as the number of days of training increased (**Figure 5C**). This suggests that daily doses of approximately 8mM (0.03 g/dl) produce the strongest cue-induced ethanol seeking (**Figure 5D**). This data is also most reminiscent of the U-shaped curve, where very low dose exposures for few days does not produce a lasting memory, moderately low dose exposures for a moderate number of days produces a strong appetitive memory, and moderately high dose exposures for many days does not produce a lasting memory.

### Ethanol Intoxication, Not Odor, Induces Cue Memories

**FIGURE 6:**
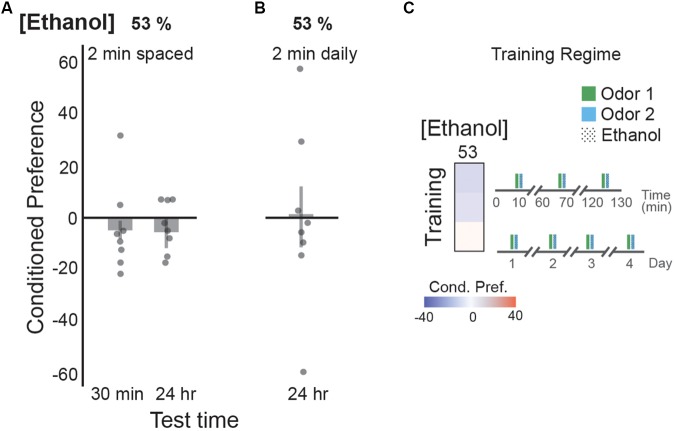
Memory is formed for ethanol intoxication, not odors. **(A)** Two minute exposure durations resulted in no preference valence with the spaced-training paradigm at 30 min, or 24 h post-training with a 53% ethanol concentration. **(B)** Single 2 min duration exposures across 4 days is not sufficient to form a cue-memory with a 53% ethanol concentration, when tested on the fifth day. Two minute exposures are not sufficient to yield increased locomotor responses that correlates with ethanol intoxication (Kaun et al., 2011). **(C)** Heat map summaries suggest no strong preference valence for either training paradigm with a 2 min exposure duration. Schematic of the training regimes are depicted. Bars represent mean +/- standard error. N∼8 per group where individual data points represent *N* = 1 (∼100 flies) CPI.

In our paradigm, flies are exposed to ethanol odor simultaneously with a neutral or appetitive odor cue. Although it is unclear whether flies can form an associative memory between two odors, 2 min training sessions with sucrose are sufficient to produce a memory for the associated cue (Schwaerzel et al., 2003; Burke et al., 2012). Importantly, 2-min exposures of 53% ethanol vapor are not sufficient to produce the locomotor stimulatory effects of ethanol (Kaun et al., 2011). Three 2-min pairings between an odor cue and 53% ethanol, spaced by 1 h rest periods produced no preference 30 min or 24 h after training (**Figure 6A**). Similarly, single 2 min pairings between 53% ethanol and an odor cue across 4 training days resulted in no odor preference (**Figure 6B**). Since similar training paradigms produced persistent memory when the ethanol exposure was long-enough to produce locomotor stimulatory effects (Kaun et al., 2011), this suggests that flies are forming memories between the pharmacological or intoxicating properties of ethanol rather than the odor of the ethanol vapor.

## Discussion

In order to further our understanding of how ethanol can co-opt the natural reward systems within the brain, it is important to understand how exposure parameters affect the reward system. Ethanol displays a dose-dependent relationship in humans that can drastically alter the displayed physiological response, and, importantly, the consumption of ethanol (Van Etten et al., 1995). Understanding how different concentrations can alter memory of the intoxication experience, and ultimately cravings, can inform our understanding of the neurobiology underlying AUD.

*Drosophila* have proven to be an effective model to study mechanisms of ethanol-induced hyper-locomotion, tolerance, reward, and sedation (Moore et al., 1998; Scholz et al., 2000; Singh and Heberlein, 2000; Wolf et al., 2002; Berger et al., 2004; Devineni and Heberlein, 2009; Kaun et al., 2011, 2012; Robinson et al., 2012; van der Linde et al., 2014; Zer et al., 2016). The genetic tractability of *Drosophila* has allowed researchers to identify genetic components underlying these ethanol-related behaviors (Heberlein et al., 2004; Kong et al., 2010; Rodan and Rothenfluh, 2010; Devineni et al., 2011). Advancements in the field have also implicated the complicated nature of ethanol-related behaviors, where an animal’s internal-state (hunger, circadian rhythm, sexual deprivation, stress, etc.) is an important factor to consider (Corl et al., 2005; van der Linde and Lyons, 2011; Shohat-Ophir et al., 2012). However, the behavioral parameters mediating the aversive or rewarding properties of ethanol memory in *Drosophila* is less understood.

In this study we provide a comprehensive characterization of a behavioral paradigm for memory of cues associated with intoxication in *Drosophila*, where we analyze the relationships between: dose concentration, number of exposure pairings, exposure duration, training paradigm, testing period, and observed preference. We found that all these factors affect the observed ethanol preference, with the most important factor being the administered dose.

### Ethanol-Dose Is a Major Determinant of Displayed Preference

In humans, there is a dose-dependent relationship between ethanol consumption and the dose concentration (Van Etten et al., 1995). Ethanol consumption increases as a function of increasing ethanol concentration. This trend continues up to a peak concentration at which point further increases in concentration result in less ethanol consumption. This inverted U-shaped relationship is conserved in rodents, where the conditioned lever-press responses increase as the dose of injected ethanol increases (Tomie et al., 1998). Once the maximum conditioned response is reached, the average lever presses decline with increasing ethanol dose (Tomie et al., 1998).

We observed that single exposure training sessions across moderate ethanol vapor concentrations display a mild trend towards an inverted U-shaped dose-preference relationship in *Drosophila* when tested 30 min post-training (**Figure 2A**). However, these memories were not long-lasting (**Figure 2B**). Similarly, we observe a mild U-shaped trend 24 h after training with a single exposure of low ethanol vapor concentrations (**Figure 2B**). The data that most resembles a U-shaped curve, however, is when flies are exposed to a single dose of ethanol vapor once a day for up to 5 days (**Figure 5**). The trend in this data suggests that doses too infrequent and too low, or conversely too frequent and too high do not produce a strong appetitive response. The ‘goldilocks’ training paradigm to produce the strongest appetitive response appears to be a dose of ethanol approximating 8mM (0.03 g/dl) body alcohol content, once a day for 4 days. Remarkably, this dose of alcohol approximates one that induces a mild euphoria in humans due to about one drink.

This dose-dependent response profile is notably similar to that seen in humans and rodents, suggesting that similar biological mechanisms may be underlying these preferences. However, it is of note that initial studies looking at these dose response relationships in humans and rodents are vastly different. The timescale across studies is not consistent, where these observations are made across weeks and months in rodent and human studies. Additionally, the methodology is vastly different. Although we are similarly looking at cue-induced responses, we are using different measures to characterize this. Whether it is *via* volitional intake of alcohol with lever-press studies in rodents, or through looking at physiological responses and cravings in clinical studies.

Understanding which ethanol concentration distinguishes between an aversive or appetitive response to an ethanol-associated cue can shape the way we understand how levels of intoxication are perceived and stored as memories. We speculate that both the immediate and long-term preferences are dose-dependent, but the level of intoxication dictates whether the flies find the associated cue appetitive or aversive. Lower concentrations are initially aversive, whereas slightly higher concentrations are sufficiently intoxicating to overcome ethanol’s aversive properties. This reflects the first exposure to ethanol being initially aversive in humans, until an association is formed between the drug and subsequent euphoria.

Our data also demonstrate that low doses of alcohol (0.025 – 0.04 g/dl body alcohol content) result in the highest appetitive memory 24 h after exposure. Despite low-dose ethanol being most behaviorally relevant in inducing alcohol preference, limited attention has been given to understanding the molecular and cellular targets of *in vivo* low-dose ethanol responses (Cui and Koob, 2017). Our results highlight the relevance of using *Drosophila* to investigate how low doses of alcohol influence the neural and molecular mechanisms underlying memory formation and behavioral decisions.

### Differences in Training Sessions Drastically Alter Choice Outcome

Training paradigms in which ethanol exposures are given in consecutive short intervals reflect the preferences that are observed in single exposure training. Flies have heightened levels of intoxication following multiple consecutive binge-like exposures (no ‘rest’ period between exposure pairings), which is rewarding shortly after training (**Figure 3**). This acute reward, however, does not induce a strong lasting memory. Intriguingly, when exposures were spaced by 1 h to allow sufficient ethanol metabolism, lower doses that were aversive shortly after training were remembered as more appetitive the following day (**Figure 4**). This is consistent with the observation that many abused substances are initially aversive until the rewarding properties are learned to be associated with the drug (Wise et al., 1976; White et al., 1977; Riley, 2011).

This stark switch in valence hints at the complex nature of how drugs of abuse may unnaturally act on the reward system. In rodents pre-exposure to ethanol conditions an appetitive memory for ethanol (Bienkowski et al., 1995; Cunningham et al., 1997; Cunningham and Henderson, 2000; Carrara-Nascimento et al., 2014). Similarly, in humans, a priming dose directly affects subsequent craving responses (Ludwig and Wikler, 1974; Hodgson et al., 1979). We speculate that this priming dose functions to initially activate circuits mediating aversion. This is later followed by the simultaneous inhibition of aversion circuits and stimulation of reward circuits inducing an enhanced appetitive response. This is affirmed by observation that single exposures of ethanol across multiple days results in stronger appetitive memories. In this case, the first day of training is a priming dose, and further activation of this reward circuitry by subsequent training increases preference.

### Relevance for Understanding Cue-Induced Cravings

Behavioral characterization in this present study highlights the similarities and differences shared across animal models in cue-induced ethanol memories. Initially, our study looks at cue-induced ethanol memory immediately following a single exposure pairing (30 min post-training) and the following day (24 h post-training) across different ethanol concentrations. As stated previously, we observe that cue-induced memory valence and strength depends on the ethanol concentration. This relationship is similar to those observations in rodent and human literature, where conditioned responses are shaped by the concentration used (Bozarth, 1990; Monti et al., 1993; Van Etten et al., 1995; Risinger and Oakes, 1996; Tomie et al., 1998; Uhl et al., 2014; Shimizu et al., 2015).

However, it is important to highlight that rodent and human studies observe these effects on a different timescale than fruit flies. Training paradigms for rodent studies typically require weeks of training, while most human studies look at patients with a history of alcohol-dependence that developed after years of alcohol abuse. Similarly, when flies are trained on a longer time scale, such as once a day for 4 days, they maintain an appetitive memory of the experience as long as the dose of alcohol is enough to be intoxicating (unlike in **Figure 6**) but not too high (as in **Figures 5A,B** but not **Figure 5C**).

Additionally, we show that concentrations that are initially found to be aversive tend to result in long-term appetitive memories. This is reminiscent of studies where ethanol is initially found to be aversive, and priming doses are used in training to elicit conditioned responses (Ludwig et al., 1974; Bienkowski et al., 1995; Cunningham et al., 1997; Cunningham and Henderson, 2000; Carrara-Nascimento et al., 2014). Interestingly, this valence switch is not observed across all concentrations and training paradigms in our study. The conditions resulting in this valence switch may be more comparable to current rodent and human studies, but training conditions that do not result in this switch may provide valuable information missing from the field. The flexibility of our behavioral system allows us to change training conditions with ease and test how different parameters result in different conditioned preferences. Thus, the behavioral flexibility provided by *Drosophila* allows us to ask questions that may be more costly in other model systems, while preserving the behavioral responses.

Our careful characterization of how ethanol concentration, timing, and number of exposures influence expression of memory for a cue associated with intoxication provides a framework for investigating the circuit, cellular and molecular mechanisms affected by low-dose ethanol exposure. This affirms the viability of *Drosophila* as a model to study mechanisms underlying cue-induced cravings at multiple levels: from molecules to single cells to network activity within a relatively complex circuit.

## Author Contributions

KK designed, performed, and analyzed the experiments. RA designed and developed the experimental apparatus. KN and KK wrote and revised the manuscript.

## Conflict of Interest Statement

The authors declare that the research was conducted in the absence of any commercial or financial relationships that could be construed as a potential conflict of interest.
